# Psychometric properties of instruments measuring ethical climate among healthcare professionals in care settings pre-pandemic: a systematic review

**DOI:** 10.1186/s12910-025-01311-4

**Published:** 2025-10-08

**Authors:** Sabine Björk, Margareta Brännström, Ulf Isaksson

**Affiliations:** Department of Nursing, Umeå University, Umeå, Sweden

**Keywords:** Ethical climate, Hospital ward, Assessment, Instrument validation studies

## Abstract

**Background:**

The ethical climate in healthcare is part of the work environment and a basis for professional nursing practice. The ethical climate is crucial as it is closely associated with staff job satisfaction, the quality-of-care provision, and nurses’ intention to stay in their current occupation and position. Even though several instruments assessing ethical climate in healthcare have been developed over the years, their psychometric properties have not been systematically reviewed.

**Objectives:**

This study was conducted to identify and critically appraise the psychometric properties of instruments used to measure the ethical climate among healthcare professionals in care settings prior to the COVID-19 pandemic.

**Methods:**

A systematic review was performed, covering papers published between 1994 and 2019, excluding grey literature sources. The literature search was performed in October 2019 in Cinahl, PsychINFO, PubMed, and SocIndex. Empirical studies were included describing the psychometric properties of instruments measuring the ethical climate among healthcare professionals in healthcare settings. Data on psychometric properties were extracted and a quality assessment was performed following the quality criteria for measurement properties proposed by Terwee et al. criteria 2007.

**Result:**

Our search yielded 15,150 publications. After title and abstract screening, 611 studies were retained for full-text analysis, of which eight studies describing five instruments were included (five instrument development studies and three translation studies). Four studies concerned the Hospital Environment Climate Scale (HECS). All instruments had been assessed for content validity and internal consistency. Information concerning criterion validity, construct validity, and reproducibility was lacking or intermediate. No information concerning floor/ceiling effect or interpretability was reported in most cases. One study reported having performed a test-retest analysis. None of the included studies fulfilled all the Terwee et al. criteria.

**Conclusion:**

Five instruments were identified as having undergone psychometric testing; however, none fulfilled all the criteria outlined by Terwee et al. Also, only one of the instruments had been subjected to the well-established test-retest analysis. This highlights a need for further well-structured validation studies of instruments assessing the ethical climate among healthcare professionals in care settings.

**Supplementary Information:**

The online version contains supplementary material available at 10.1186/s12910-025-01311-4.

## Background

A healthy work environment in healthcare is a place of physical, mental, and social well-being where all healthcare professionals can foster the health and safety of their patients and colleagues. Therefore, the work environment needs to cultivate a sense of security, respect, and empowerment for all multidisciplinary team members. The ethical climate is part of the work environment, embracing staff-shared perceptions of moral behaviour concerning ethical issues [1]. The term “ethics” can be understood as a broad concept that encompasses the understanding and examination of moral life through both normative and non-normative approaches [2]. According to the classical definition by Victor and Cullen (1987) [3], an ethical climate is described as a set of shared perceptions regarding procedures and policies, both formal and informal, which shape expectations for ethical behaviour within an organisation or company. A concept analysis [4] defined the ethical climate as “a process articulating several concepts and elements regarding the organisational aspect, ethics, and the workers’ well-being”. A systematic review found variability of ethical climate in healthcare, as measured by the Ethical Climate Questionnaire (ECQ), the Hospital Ethical Climate Survey (HECS) and the Environment Questionnaire (EEQ) [5]. The analysis found no study and sample-related characteristics that explained this heterogeneity.

A positive ethical climate in hospital organisations is considered a key factor since it has been associated with staff job satisfaction[6, 7], moral distress[8–11], general well-being[9], quality of care provision[12, 13], and nurses’ intention to stay in their current position/occupation[9, 13]. Instruments assessing moral issues, such as the Moral Distress Scale [14] and Moral Sensitivity Questionnaire[15], have often been used. However, these instruments assess ethics at an individual level and the individual’s moral or ethical reasoning, not the ethical climate at a group level. Ethical competence is a crucial component of nursing[16], and the associated ethical climate is the basis for professional nursing practice [9] and it may impact health services delivery, access and patient safety[9, 12, 13]. A valid ethical climate assessment can help identify areas for improvement and evaluate interventions. Unfortunately, although instruments for assessing ethical climate in healthcare have been developed over the years, their development and measurement properties have not been systematically reviewed.

### Aim

To identify and critically appraise the psychometric properties of instruments used to measure the ethical climate among healthcare professionals prior to the COVID-19 pandemic.

## Method

A systematic literature review was chosen for this study as it is a useful method for obtaining an overview of the available instruments for measuring ethical climate and their psychometric properties. This systematic review applied the Preferred Reporting Items for Systematic Reviews and Meta-Analyses (PRISMA) guidelines[17].

### Search strategy

We conducted a systematic literature review following an a-priori-defined, unregistered protocol. The systematic literature searches were performed in October 2019 in the Cinahl, PsycINFO, PubMed, and Socindex electronic databases. The systematic database search was designed and performed by two librarians with expertise in healthcare literature, supported by input from the research team, which had expertise in care ethics and conducting systematic reviews. The search strategy focused on the concepts “Ethic or Moral”, “Instrument”, and “Healthcare personnel” and their corresponding index terms with synonyms (see Appendix Supplementary 1 for details).

### Eligibility criteria

We included studies published from January 1994 to September 2019 in English (excluding grey papers) describing empirical studies, where the psychometric properties of instruments measuring the ethical climate among healthcare professionals in care settings were evaluated.

Papers excluded were all types of reviews, case reports, editorials, short communication, protocols, non-empirical (theoretical and discussion).

### Study selection

The studies were selected in two phases. First, all search hits were inserted into Rayyan software [18], and duplicates were removed. Following this, the search hits were equally divided among four pairs of reviewers who independently screened their titles and abstracts for congruence with the inclusion criteria – an instrument measuring ethical and/or moral climate, where the participants were healthcare professionals, the context was the care setting, and the studies were published in English. After this, articles were inserted into EndNote, and full-text copies were retrieved and screened. Each step was performed independently, after which the independent review process results were compared in the pairs. Any discrepancies were discussed in the pairs until a consensus was reached. In the second phase, the final full-text articles were independently reviewed by the authors SB, MB, and UI to assess eligibility criteria for inclusion in this review and identify studies that had psychometrically tested a relevant instrument; the results were then compared, and discrepancies were resolved through discussion until consensus was reached among the authors SB, MB and UI. 

### Data extraction, quality assessment, and measurement properties

The data extracted in this review were the following: authors, publication year, and publication country; full instrument name, language, number of items, subscales, dimensions, and response options; theoretical background; participant characteristics in terms of age, female/male ratio, and population; sample size; measurement properties in terms of content validity, internal consistency, criterion validity, construct validity, agreement, reliability, responsiveness, floor/ceiling effect, and interpretability; and results of exploratory and confirmatory factor analyses [19]. The data from the included articles were compiled in Tables 1, 2 and 3, and 4 in order to describe all the instruments and their psychometric properties as well as the analyses performed, for example: exploratory factor analysis (EFA), confirmatory factor analysis (CFA), and Cronbach’s alpha. These results were then compared to the applicable thresholds [28–31]. A quality assessment of each scale was performed using the Terwee et al.[32] criteria (Appendix Supplementary 2). A rating of “+” indicates a clear description, “?” a doubtful design or method, and “0” for no information found.

**Table 1:** Characteristics of included studies in alphabetical order

Author	Instrument	Language	Sample characteristics		
			*n*	Age, mean (SD)	Female %
Borhani et al. (2014) [20]	Ethical Climate Questionnaire (ECQ)	Farsi	275 nurses		
Charalambous et al. (2018) [21]	Hospital Ethical Climate Survey (HECS)	Greek	235 nurses		84.3
Grönlund et al. (2019) [22]	Swedish Ethical Climate Questionnaire (SwECQ)	Swedish & English	355 healthcare workers		85.4
Khalesi et al. (2014) [23]	Hospital Ethical Climate Survey (HECS)	Persian	187 nurses		89.9
McDaniel (1997) [24]	Ethics Environment Questionnaire (EEQ)	English	450 nurses		
Olson (1998) [25]	Hospital Ethical Climate Survey (HECS)	English	360 nurses		95.0
Van Den Bulcke et al. (2018) [26]	Ethical Decision-Making Climate Questionnaire (EDMCQ)	English	3610 nurses and 2275 physicians	38.0 (30.0–47.0)	
Suhonen et al. (2015) [27]	Hospital Ethical Climate Survey (HECS-S)	Finnish	874 nurses	42 (± 12.6)	95.0

**Table 2:** Characteristics of included instruments

Instrument	Reference	Number of items	Subscales	Domains	Response options	Theoretical background
ECQ	Borhani et al. (2014) [20]	26	5	Caring, rules, instrumental, professionalism, and independence	5-point Likert scale	
EEQ	McDaniel (1997) [24]	20	Uni-dimensional		5-point Likert scale	
HECS	Khalesi et al. (2014) [23], Olson (1998) [25], Suhonen et al. (2015) [27], and Charalambous et al. (2018) [21]	26	5	Managers, physicians, peers, hospital, and patients	5-point Likert scale	
SwECQ	Grönlund et al. (2019) [22]	12	Uni-dimensional		6-point Likert scale	Habermas
EDMCQ	Van Den Bulcke et al. (2018) [26]	32	7	Self-reflective and empowering leadership by physicians; practice and culture of open interdisciplinary reflection; culture of not avoiding end-of-life decisions; culture of mutual respect within the interdisciplinary team; active involvement of nurses in end-of-life care and decision-making; active decision-making by physicians; and practice and culture of ethical awareness	4- and 5-point Likert scales	

**Table 3:** Psychometric properties of instruments included in the study

Author, year	Scale	Reliability	Content validity	EFA	CFA goodness-of-fit	Inter-correlation
Borhani et al. (2014) [20]	ECQ	α subscales: 0.711–0.758				
Charalambous et al. (2018) [21]	HECS	α tot: 0.875 α subscales: 0.672–0.736				
Grönlund et al. (2019) [22]	SwECQ	α tot: 0.86 α subscales: 0.75–0.82	S-CVI = 0.88 I-CVI = 0.8–1.0	Unidimensional 4 factor F1 (4 items, 0.49–0.71), F2 (1 item, 0.39), F3 (3 items, 0.51–0.80), F4 (2 items, 0.41–0.97)	C_min_/df: 3.07 *p* < 0.001 TLI: 0.92 NFI: 0.93 CFI: 0.95 RMSEA: 0.08	0.507–0.752
Khalesi et al. (2014) [23]	HECS	α tot: 0.94 α subscales: 0.69–0.85			C_min_/df: 1.99 *p* < 0.001 CFI: 0.96 SRMR: 0.064 NNFI: 0.96 RMSEA: 0.075	
Olson (1998) [25]	HECS	α tot: 0.91 α subscales: 0.68–0.92	CVI = 0.89	F1 (4 items, 0.63–0.79), F2 (4 items, 0.47–0.74), F3 (6 items, 0.80–0.90), F4 (6 items, 0.56–0.73), F5 (6 items, 0.51–0.85)	C_min_/df: 1.39 AGFI: 0.95 RMR: 0.06	
Van Den Bulcke et al. (2018) [26]	EDMCQ	α tot: 0.897		F1 (7 items, 0.43–0.71), F2 (7 items, 0.37–0.70), F3 (4 items, 0.41–0.78), F4 (3 items, 0.41–0.69), F5 (3 items, 0.54–0.78), F6 (4 items, 0.39–0.63), F7 (4 items, 0.54–0.57) 46% explained variation	CFI: 0.891 AGFI: 0.897 RMSEA: 0.053 SRMR: 0.048 AIC: 208 986.7	
Suhonen et al. (2015) [27]	HECS-S	α tot: 0.92 α subscales: 0.58–0.92		60.7% explained variation	RMSEA: 0.13 GFI: 0.96 CFI: 0.97 RMR: 0.016 SRMR: 0.041	
McDaniel (1997) [24]	EEQ	α tot; 0.93 Test–retest: 0.88	Face validity	Unidimensional 0.38		0.38–0.77

**Table 4:** Summary of the assessment of the measurement properties of all questionnaires measuring ethical climate

Questionnaire	Author	Content validity	Internal consistency	Criterion validity	Construct validity	Reproducibility				
						Agreement	Reliability	Responsiveness	Floor/ceiling effect	Interpretability
ECQ	Borhani et al. (2014) [20]	+	+	0	?	0	0	0	0	?
EEQ	McDaniel (1997) [24]	+	+	+	?	+	0	+	0	0
HECS	Charalambous et al. (2018) [21]	+	+	0	0	0	0	0	0	0
HECS	Khalesi et al. (2014) [23]	+	+	0	0	0	0	0	?	?
HECS	Olson (1998) [25]	+	+	0	+	0	0	0	0	0
HECS-S	Suhonen et al. (2015) [27]	+	+	0	0	0	?	0	0	+
SwECQ	Grönlund et al. (2019) [22]	+	+	0	0	0	0	0	–	0
EDMCQ	Van Den Bulcke et al. (2018) [26]	+	+	?	0	0	?	0	0	0

Rating: + = positive,? = Intermediate, - = Poor; 0 = No information available

## Results

### Study selection

**Fig. 1:**
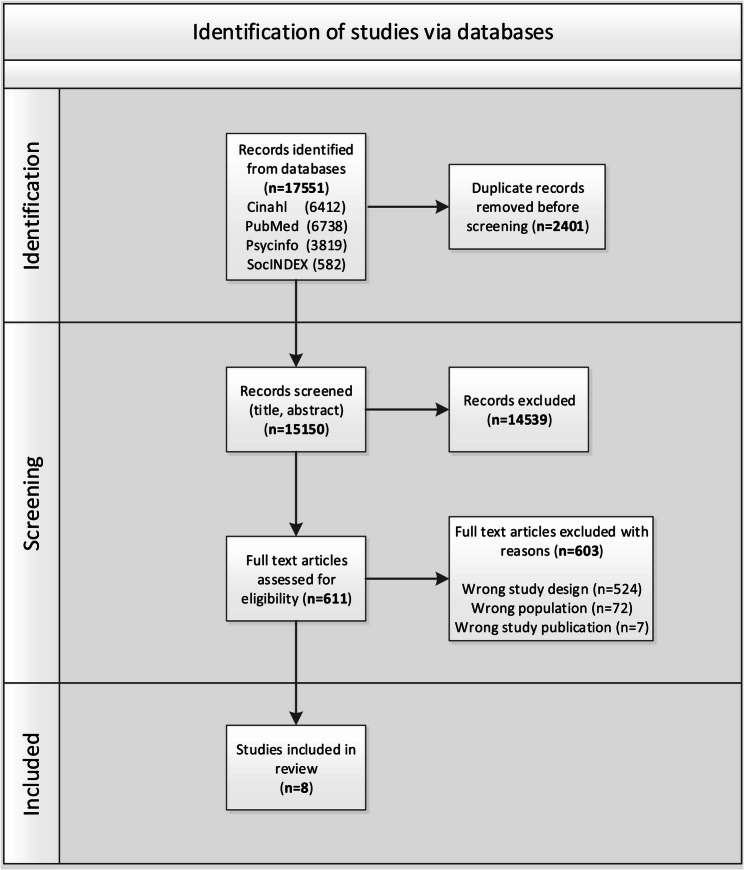
PRISMA flowchart of study selection

The search identified 17,551 potential articles from four databases. After removing duplicates (*n* = 2401) and screening titles and abstracts, 611 full-text articles were assessed for eligibility. Eight articles met the inclusion criteria and were included in this review. Most articles excluded at the full-text level were excluded because of the wrong study design (*n* = 524). The flow diagram of the study selection is presented in Fig. 1.

### Study characteristics

Eight studies of instruments assessing ethical climate were included (Table 1); four of these were instrument development studies, and four were instrument translation studies. The number of participants in the studies ranged from 187 to 5885.

### Instruments included

In total, five instruments assessing ethical climate were described: the Ethical Climate Questionnaire (ECQ)[20], the Ethics Environment Questionnaire (EEQ)[24], the Hospital Ethical Climate Survey (HECS)[21, 23, 25, 27, 33], the Swedish Ethical Climate Questionnaire (SwECQ)[22], and the Ethical Decision-Making Climate Questionnaire (EDMCQ) [26]. Most of the included studies examined HECS. All the included studies were published in 2014 and later, except for two [24, 25] published in 1997 and 1998, respectively. The instruments were translated into six languages: English, *n* = 3; Farsi, *n* = 1; Persian, *n* = 1; Greek, *n* = 1; Finnish, *n* = 1; and Swedish, *n* = 1 (the SwECQ was presented in two languages).

### Descriptions of the instruments

The number of items per scale ranged from 12 (SwECQ) to 32 (EDMCQ), with a median of 26 items per scale. All items were responded to on a 5-point Likert scale, except for one instrument (SwECQ), which used a 6-point Likert scale. EDMCQ used a combination of 4- or 5-point Likert scales. All scales consisted of five dimensions, except for EDMCQ, which had seven dimensions, and SwECQ and EEQ, which were considered unidimensional. Five studies [20–22, 24, 27] presented how to score the scale, while three did not. Olson [25] did not specify how the scoring would be done in her original article.

### Psychometric properties

All instruments had been assessed for content validity and internal consistency. Information concerning criterion validity, construct validity, and reproducibility was lacking or intermediate. No information concerning the floor/ceiling effect or interpretability was reported in most cases. One study reported having performed a test-retest analysis [24]. 

For all instruments besides the Swedish version of HECS-S[33], Cronbach’s alphas were presented. The alphas for the other instruments varied somewhat but were relatively high, i.e., 0.86–0.94; the respective subscales had lower alphas, i.e., 0.61–0.94. Three studies explored content validity. Two studies [22, 24] analysed the content validity index for SwECQ and HECS. The other studies did not report any content validity test, except for the one by McDaniel[24], which used face validity. Five studies [22, 24–27] reported having conducted an explorative factor analysis. Five studies had developed a new scale, while three translated earlier scales. Five studies [22, 24–27] reported having conducted goodness-of-fit testing, while two did not [20, 23]. Two studies [22, 24] reported some inter-correlation.

### Quality assessment and measurement properties

A quality assessment of each scale was performed using the Terwee et al.[32] criteria (Appendix Supplementary 2). The quality assessment found that all instruments had been content-validated and tested for internal consistency. However, the studies did not include criterion validity, construct validity and reproducibility. The same applied to responsiveness, floor/ceiling effect and interpretability (Table 4).

## Discussion

This review showed that there are relatively few instruments measuring ethical climate. This review found only five psychometrically tested instruments assessing the ethical climate in hospital wards. Two instruments were formulated in the 1990 s; however, interest in assessing the ethical climate has increased as more instruments have been developed recently. One instrument (EEQ [24]) measured the ethical climate unidimensionally, while another (SwECQ [22]) assessed the climate unidimensionally but could also measure it in four dimensions. All the other instruments assessed the climate in five dimensions, except for EDMCQ[26], which assessed the climate in seven domains.

All the original instruments had stated satisfactory validity; however, some of the translated instruments [20, 33] had not undergone any validity or reliability testing. Most instruments had been subject to EFA and CFA for validation. Such exploratory analyses are appropriate in a newly developed instrument when the dimensionality is unknown. However, as shown in Table 3, five studies used CFA for analysis. Of these, two involved translated versions, while three were original developed instruments. With a well-established version of an instrument or a translation, a CFA or IRT may be appropriate [34]; however, it may be more questionable why the development of an instrument has used CFA. The reliability of all instruments was measured using Cronbach’s alpha, showing high internal homogeneity between the different items. However, none of the instruments had been subjected to concurrent validity testing, possibly due to the lack of instruments assessing the ethical climate. These instruments should be further refined or translated into new languages. However, translating an instrument too easily from one language to another can be precarious. It is not just verbatim translation that can cause difficulties; it is also the case that there may be a completely different culture and healthcare system in a country other than where the instrument was initially developed, which may mean that items can be interpreted differently from how they were originally intended [35]. Thus, a translated instrument may not always be suitable for a context other than where it has been developed. It is difficult to say what instrument should be used as the “gold standard”. As HECS is the oldest and most widely used instrument, having been translated into several languages, it could be regarded as an instrument for comparison.

Interestingly, only the original EEQ [24] had been subjected to test-retest analysis to assess the instrument’s consistency over time. However, the retest was conducted four months after the previous test, which could be regarded as an excessive inter-test interval, preferably only 2–4 weeks [36]. Nevertheless, since none of the instruments has been tested over time, it can be questioned whether any change in the results is a real change over time due to, for example, a staged intervention or whether it is caused by the instrument not being stable over time. It is, therefore, essential to conduct a test-retest analysis of whichever instrument is preferred before using it in an intervention. Ethical climate is an area that has been insufficiently explored, and, only a few instruments have measured it. The oldest and most translated scale, and thus apparently the most widely used, is HECS, which can currently be seen as a standard for measuring the ethical climate in hospital wards. It seems as the instruments included in this systematic review should be subjected to ongoing research and development. Especially important is test-retest analysis, as it is still unclear whether these scales remain stable over time, i.e., whether any detected change over time represents a change in the ethical climate (e.g., due to an intervention) or is simply because the instrument is not stable over time. The COSMIN guidelines for methodological quality were introduced in 2010, and two of the studies [24, 25] included in this review were published before that time. If supported by these guidelines, the authors of those studies might have made other study design choices, such as conducting test-retest analysis.

### Methodological considerations

Strengths of this review include the systematic electronic search strategy, which a librarian developed in collaboration with the authors, and the independent assessment of eligible studies. This review included studies available in full text and published in English. Consequently, studies in other languages or the grey literature describing measurement properties and scale development were not included, which may have led to the exclusion of relevant research. However, the most important studies will likely be found in established databases.

An unbiased systematic review can aid in instrument selection. A strength of this study is the use of the quality criteria for measurement properties proposed by Terwee et al.,[32] ensuring that all studies were systematically assessed based on the same criteria. However, as none of the included studies fulfilled all the quality criteria, it is up to the readers of this review to decide which instrument best suits their purpose. The limited number of psychometric tests also highlights a gap and, thus, a need for further validation studies.

One could argue that this study is limited since we only included studies pre-pandemic. The COVID-19 pandemic constituted an acute crisis within healthcare. It is conceivable that the experiences faced by healthcare personnel during the pandemic have led to a shift in perceptions of what the ethical climate entails. Consequently, it is possible that newly developed instruments now measure aspects of the ethical climate that differ from those assessed prior to the pandemic. This is an area that future research needs to address.

## Conclusion

The psychometric properties of instruments assessing ethical climate among care professionals in care settings have been identified and reviewed in this review. Although five instruments were identified as having undergone psychometric testing, none fulfilled all the criteria outlined by Terwee et al.[32] Also, only one of the instruments had been subjected to the well-established test-retest analysis. This highlights a need for further well-structured validation studies of instruments assessing the ethical climate among healthcare professionals in care settings.

We have difficulties in giving any recommendations on how to improve ethical climate to healthcare policymakers or healthcare workers since this study has a focus on the psychometric properties of the different instruments - not the direct results based on them. However, we recommend stakeholders to choose carefully an instrument that is psychometrically sound and that has been used in the past in order to be able to assess more easily whether there have been any changes in the ethical climate. Choosing the proper questionnaire for a specific purpose can be time-consuming and difficult. A systematic review, such as this one, can serve as a tool to support evidence-based instrument selection.

## Supplementary Information

Supplementary Material 1

Supplementary Material 2

## Data Availability

All data generated or analyzed during this review are included in this published article and its supplementary material. The articles reviewed in this systematic review are available in the associated journals in which they were originally published. Full citation details of these articles are included in the reference section. The search strings used to generate the datasets can be found in the supplementary information files.
