# Supplementary material for: Psychometric properties of instruments measuring ethical climate among healthcare professionals in care settings pre-pandemic: a systematic review
Source: BMC Med Ethics. 2025 Oct 8;26:125. doi: 10.1186/s12910-025-01311-4 (PMC12506347; doi:10.1186/s12910-025-01311-4)
Supplement: Supplementary file 1 — Supplementary Material 1 [file 12910_2025_1311_MOESM1_ESM.docx]

**Supplemental material 1. Search strategies for all resources**

## [CINAHL](http://proxy.ub.umu.se/login?url=http://search.epnet.com/login.asp?profile=web&defaultdb=c8h)

### S1 Ethical/moral aspects

(MH "Ethics+") OR TI ethic* OR IN (ethic* OR moral OR morals OR morally OR morality OR virtue OR virtues OR conscience OR "professional code" OR "professional codes" OR "professional value" OR "professional values") OR (moral OR morals OR morally OR morality OR virtue OR virtues OR conscience OR "professional ethic" OR "professional ethics" OR "clinical ethic" OR "clinical ethics" OR "medical ethic" OR "medical ethics" OR "dental ethic" OR "dental ethics" OR "nursing ethic" OR "nursing ethics" OR "pharmaceutical ethic" OR "pharmaceutical ethics" OR "pharmacy ethic" OR "pharmacy ethics" OR "ethical analysis" OR "ethical analyses" OR "ethical climate" OR "ethical competence" OR "workplace ethic" OR "workplace ethics" OR "organizational ethic" OR "organizational ethics" OR "organisational ethic" OR "organisational ethics" OR "ethical dilemma" OR "ethical dilemmas" OR "ethical difficulty" OR "ethical difficulties" OR "ethical conflict" OR "ethical conflicts" OR "ethical environment" OR "ethical environments" OR "ethical practice" OR "ethical practices" OR "ethical issue" OR "ethical issues" OR "professional code" OR "professional codes" OR "professional value" OR "professional values") NOT (MH "Research Ethics+" OR MH "Animals, Laboratory")

AND

### S2 Healthcare professionals

(MH "Health Personnel+" OR MH "Attitude of Health Personnel+" OR "health personnel" OR "healthcare personnel" OR "care personnel" OR "medical personnel" OR "care provider" OR "care providers" OR "healthcare provider" OR "healthcare providers" OR "care worker" OR "care workers" OR "healthcare worker" OR "healthcare workers" OR "health worker" OR "health workers" OR "care professional" OR "care professionals" OR "healthcare professional" OR "healthcare professionals" OR "health professional" OR "health professionals" OR "healthcare team" OR "healthcare teams" OR "care team" OR "care teams" OR "health employee" OR "health employees" OR "hospital staff" OR "medical staff" OR "healthcare staff" OR "care staff" OR "health staff" OR "hospital personnel" OR nurse OR nurses OR "nursing staff" OR "nursing team" OR "nursing teams" OR midwife OR midwives OR physician OR physicians OR allergists OR anesthesiologists OR cardiologists OR dermatologists OR endocrinologists OR gastroenterologists OR "general practitioners" OR geriatricians OR hospitalists OR nephrologists OR neurologists OR oncologists OR ophthalmologists OR otolaryngologists OR pathologists OR pediatricians OR physiatrists OR pulmonologists OR radiologists OR rheumatologists OR surgeons OR urologists OR neonatologists OR neurosurgeons OR psychiatrists OR clinicians OR doctors OR dentists OR anesthetists OR "healthcare practitioner" OR "healthcare practitioners" OR "health practitioner" OR "health practitioners" OR "care practitioner" OR "care practitioners" OR "nursing assistant" OR "nursing assistants" OR "home health aide" OR "home health aides" OR "home care aide" OR "home care aides" OR "healthcare assistants" OR "care assistants" OR "health assistants" OR therapists OR physiotherapists OR dieticians OR dietitians OR nutritionists OR "dental hygienists" OR "dental staff" OR paramedic OR paramedics OR "paramedical personnel" OR "healthcare support workers" OR "social workers" OR psychologists OR psychotherapists OR "psychiatric aides") NOT (MH "Veterinary Medicine+")

AND

### S3 Measuring instrument

(MH "Questionnaires+" OR MH "Psychometrics" OR MH "Reliability and Validity+" OR MH "Scales" OR MH "Instrument Validation" OR MH "Instrument Construction+" OR MH "Instrument Scaling+" OR MH "Surveys" OR MH "Validation Studies" OR MH "Factor Analysis" OR surveys OR survey OR questionnaires OR questionnaire OR instrument OR instruments OR scale OR scales OR screening OR validation OR validity OR validated OR "internal consistency" OR reproducibility OR reliability OR responsiveness OR "ceiling effects" OR "factor analysis" OR "factor analyses" OR psychometric OR psychometrics)

### S4

S1 AND S2 AND S3

Result: 9,888

### S5 Filter

Limiters: 1994-, english, swedish. Source types: academic journals

Result: 6,412

## [PsycINFO](http://proxy.ub.umu.se/login?qurl=http%3A//search.ebscohost.com/login.aspx%3Fauthtype%3Dip%2Cuid%26profile%3Dehost%26defaultdb%3Dpsyh)

### S1 Ethical/moral aspects

(DE "Morality" OR DE "Ethics" OR DE "Professional Ethics" OR TI ethic* OR TM ethic* OR moral OR morals OR morally OR morality OR virtue OR virtues OR conscience OR "professional ethic" OR "professional ethics" OR "clinical ethic" OR "clinical ethics" OR "medical ethic" OR "medical ethics" OR "dental ethic" OR "dental ethics" OR "nursing ethic" OR "nursing ethics" OR "pharmaceutical ethic" OR "pharmaceutical ethics" OR "pharmacy ethic" OR "pharmacy ethics" OR "ethical analysis" OR "ethical analyses" OR "ethical climate" OR "ethical competence" OR "workplace ethic" OR "workplace ethics" OR "organizational ethic" OR "organizational ethics" OR "organisational ethic" OR "organisational ethics" OR "ethical dilemma" OR "ethical dilemmas" OR "ethical difficulty" OR "ethical difficulties" OR "ethical conflict" OR "ethical conflicts" OR "ethical environment" OR "ethical environments" OR "ethical practice" OR "ethical practices" OR "ethical issue" OR "ethical issues" OR "professional code" OR "professional codes" OR "professional value" OR "professional values") NOT (DE "Animal Research" OR DE "Animal Welfare")

AND

### S2 Healthcare professionals

(DE "Health Care Services" OR DE "Health Personnel" OR DE "Allied Health Personnel" OR DE "Caregivers" OR DE "Medical Personnel" OR DE "Mental Health Personnel" OR DE "Therapists" OR DE "Occupational Therapists" OR DE "Physical Therapists" OR DE "Psychotherapists" OR DE "Speech Therapists" OR DE "Clinicians" OR DE "Psychologists" OR DE "Social Workers" OR DE "Psychiatric Social Workers" OR DE "Rehabilitation Counselors" OR DE "Health Personnel Attitudes" OR "health personnel" OR "healthcare personnel" OR "care personnel" OR "medical personnel" OR "care provider" OR "care providers" OR "healthcare provider" OR "healthcare providers" OR "care worker" OR "care workers" OR "healthcare worker" OR "healthcare workers" OR "health worker" OR "health workers" OR "care professional" OR "care professionals" OR "healthcare professional" OR "healthcare professionals" OR "health professional" OR "health professionals" OR "healthcare team" OR "healthcare teams" OR "care team" OR "care teams" OR "health employee" OR "health employees" OR "hospital staff" OR "medical staff" OR "healthcare staff" OR "care staff" OR "health staff" OR "hospital personnel" OR nurse OR nurses OR "nursing staff" OR "nursing team" OR "nursing teams" OR midwife OR midwives OR physician OR physicians OR allergists OR anesthesiologists OR cardiologists OR dermatologists OR endocrinologists OR gastroenterologists OR "general practitioners" OR geriatricians OR hospitalists OR nephrologists OR neurologists OR oncologists OR ophthalmologists OR otolaryngologists OR pathologists OR pediatricians OR physiatrists OR pulmonologists OR radiologists OR rheumatologists OR surgeons OR urologists OR neonatologists OR neurosurgeons OR psychiatrists OR clinicians OR doctors OR dentists OR anesthetists OR "healthcare practitioner" OR "healthcare practitioners" OR "health practitioner" OR "health practitioners" OR "care practitioner" OR "care practitioners" OR "nursing assistant" OR "nursing assistants" OR "home health aide" OR "home health aides" OR "home care aide" OR "home care aides" OR "healthcare assistants" OR "care assistants" OR "health assistants" OR therapists OR physiotherapists OR dieticians OR dietitians OR nutritionists OR "dental hygienists" OR "dental staff" OR paramedic OR paramedics OR "paramedical personnel" OR "healthcare support workers" OR "social workers" OR psychologists OR psychotherapists OR "psychiatric aides") NOT (DE "Veterinary Medicine")

AND

### S3 Measuring instrument

(DE "Measurement" OR DE "Professional Measures" OR DE "Psychometrics" OR DE "Screening" OR DE "Testing" OR DE "Test Construction" OR DE "Questionnaires" OR DE "Surveys" OR DE "Factor Analysis"OR surveys OR survey OR questionnaires OR questionnaire OR instrument OR instruments OR scale OR scales OR screening OR validation OR validity OR validated OR "internal consistency" OR reproducibility OR reliability OR responsiveness OR "ceiling effects" OR "factor analysis" OR "factor analyses" OR psychometric OR psychometrics)

### S4

S1 AND S2 AND S3

Result: 5,716

### S5 Filter

Limiters: 1994-, english, swedish. Source types: academic journals

Result: 3,819

## PubMed

### [S1 Ethical/moral aspects](https://www.ncbi.nlm.nih.gov/mesh/68009014)

("morals"[mesh terms] OR moral[tiab] OR morals[tiab] OR morally[tiab] OR morality[tiab] OR virtue[tiab] OR virtues[tiab] OR conscience[tiab] OR ethic[ti] OR ethics[ti] OR ethical[ti] OR ethically[ti] OR "professional ethic"[tiab] OR "professional ethics"[tiab] OR "clinical ethic"[tiab] OR "clinical ethics"[tiab] OR "medical ethic"[tiab] OR "medical ethics"[tiab] OR "dental ethic"[tiab] OR "dental ethics"[tiab] OR "nursing ethic"[tiab] OR "nursing ethics"[tiab] OR "pharmaceutical ethic"[tiab] OR "pharmaceutical ethics"[tiab] OR "pharmacy ethic"[tiab] OR "pharmacy ethics"[tiab] OR "ethical analysis"[tiab] OR "ethical analyses"[tiab] OR "ethical climate"[tiab] OR "ethical competence"[tiab] OR "workplace ethic"[tiab] OR "workplace ethics"[tiab] OR "organizational ethic"[tiab] OR "organizational ethics"[tiab] OR "organisational ethic"[tiab] OR "organisational ethics"[tiab] OR "ethical dilemma"[tiab] OR "ethical dilemmas"[tiab] OR "ethical difficulty"[tiab] OR "ethical difficulties"[tiab] OR "ethical conflict"[tiab] OR "ethical conflicts"[tiab] OR "ethical environment"[tiab] OR "ethical environments"[tiab] OR "ethical practice"[tiab] OR "ethical practices"[tiab] OR "ethical issue"[tiab] OR "ethical issues"[tiab] OR "professional code" [tiab] OR "professional codes" [tiab] OR "professional value"[tiab] OR "professional values"[tiab]) NOT ("animal experimentation"[mesh terms] OR "ethics, research"[majr] OR "ethics committees, research"[majr])

### [S2 Healthcare professionals](https://www.ncbi.nlm.nih.gov/mesh/68006282)

("health personnel"[mesh terms] OR "attitude of health personnel"[mesh terms] OR "health personnel"[tiab] OR "healthcare personnel"[tiab] OR "care personnel"[tiab] OR "medical personnel"[tiab] OR "care provider"[tiab] OR "care providers"[tiab] OR "healthcare provider"[tiab] OR "healthcare providers"[tiab] OR "care worker"[tiab] OR "care workers"[tiab] OR "healthcare worker"[tiab] OR "healthcare workers"[tiab] OR "health worker"[tiab] OR "health workers"[tiab] OR "care professional"[tiab] OR "care professionals"[tiab] OR "healthcare professional"[tiab] OR "healthcare professionals"[tiab] OR "health professional"[tiab] OR "health professionals"[tiab] OR "healthcare team"[tiab] OR "healthcare teams"[tiab] OR "care team"[tiab] OR "care teams"[tiab] OR "hospital staff"[tiab] OR "medical staff"[tiab] OR "healthcare staff"[tiab] OR "care staff"[tiab] OR "health staff"[tiab] OR "health employee"[tiab] OR "health employees"[tiab] OR "hospital personnel"[tiab] OR nurse[tiab] OR nurses[tiab] OR "nursing staff"[tiab] OR "nursing team"[tiab] OR "nursing teams"[tiab] OR midwife[tiab] OR midwives[tiab] OR physician[tiab] OR physicians[tiab] OR allergists[tiab] OR anesthesiologists[tiab] OR cardiologists[tiab] OR dermatologists[tiab] OR endocrinologists[tiab] OR gastroenterologists[tiab] OR "general practitioners"[tiab] OR geriatricians[tiab] OR hospitalists[tiab] OR nephrologists[tiab] OR neurologists[tiab] OR oncologists[tiab] OR ophthalmologists[tiab] OR otolaryngologists[tiab] OR pathologists[tiab] OR pediatricians[tiab] OR physiatrists[tiab] OR pulmonologists[tiab] OR radiologists[tiab] OR rheumatologists[tiab] OR surgeons[tiab] OR urologists[tiab] OR neonatologists[tiab] OR neurosurgeons[tiab] OR psychiatrists[tiab] OR clinicians[tiab] OR doctors[tiab] OR dentists[tiab] OR anesthetists[tiab] OR "healthcare practitioner"[tiab] OR "healthcare practitioners"[tiab] OR "health practitioner"[tiab] OR "health practitioners"[tiab] OR "care practitioner"[tiab] OR "care practitioners"[tiab] OR "nursing assistants"[tiab] OR "nursing assistants"[tiab] OR "home health aide"[tiab] OR "home health aides"[tiab] OR "home care aide"[tiab] OR "home care aides"[tiab] OR "healthcare assistants"[tiab] OR "care assistants"[tiab] OR "health assistants"[tiab] OR therapists[tiab] OR physiotherapists[tiab] OR dieticians[tiab] OR dietitians[tiab] OR nutritionists[tiab] OR dentists[tiab] OR "dental staff"[tiab] OR "paramedics"[tiab] OR "paramedical personnel"[tiab] OR "healthcare support workers"[tiab] OR "social worker"[tiab] OR "social workers"[tiab] OR psychologists[tiab] OR psychotherapists[tiab] OR paramedics[tiab] OR "psychiatric aides"[tiab]) NOT "veterinarians"[mesh terms]

### [S3 Measuring instrument](https://www.ncbi.nlm.nih.gov/mesh/68011795)

"surveys and questionnaires"[mesh terms] OR "validation studies"[pt] OR "psychometrics"[mesh terms] OR "reproducibility of results"[mesh terms] OR "factor analysis, statistical"[mesh terms] OR surveys[tiab] OR survey[tiab] OR questionnaires[tiab] OR questionnaire[tiab] OR instrument[tiab] OR instruments[tiab] OR scale[tiab] OR scales[tiab] OR screening[tiab] OR validation[tiab] OR validity[tiab] OR validated[tiab] OR "internal consistency"[tiab] OR reproducibility[tiab] OR reliability[tiab] OR responsiveness[tiab] OR "ceiling effects"[tiab] OR "factor analysis"[tiab] OR "factor analyses"[tiab] OR psychometric[tiab] OR psychometrics[tiab]

### S4

S1 AND S2 AND S3

Result: 8.450

### S5 Filter

NOT ("animals"[mesh terms] NOT "humans"[mesh terms])

NOT ("study protocol"[ti] OR letter[pt] OR comment[pt] OR editorial[pt])

AND (english[lang] OR swedish[lang])

AND ("1994/01/01"[PDAT] : "3000/12/31"[PDAT])

Result: 6.738

## [SocINDEX](http://proxy.ub.umu.se/login?qurl=http%3A//search.ebscohost.com/login.aspx%3Fauthtype%3Dip%2Cuid%26profile%3Dehost%26defaultdb%3Dsnh)

### S1 Ethical/moral aspects

TI ( ethic* OR moral OR morals OR morally OR morality OR virtue OR virtues OR conscience OR "professional ethic" OR "professional ethics" OR "clinical ethic" OR "clinical ethics" OR "medical ethic" OR "medical ethics" OR "dental ethic" OR "dental ethics" OR "nursing ethic" OR "nursing ethics" OR "pharmaceutical ethic" OR "pharmaceutical ethics" OR "pharmacy ethic" OR "pharmacy ethics" OR "ethical analysis" OR "ethical analyses" OR "ethical climate" OR "ethical competence" OR "workplace ethic" OR "workplace ethics" OR "organizational ethic" OR "organizational ethics" OR "organisational ethic" OR "organisational ethics" OR "ethical dilemma" OR "ethical dilemmas" OR "ethical difficulty" OR "ethical difficulties" OR "ethical conflict" OR "ethical conflicts" OR "ethical environment" OR "ethical environments" OR "ethical practice" OR "ethical practices" OR "ethical issue" OR "ethical issues" OR "professional code" OR "professional codes" OR "professional value" OR "professional values" ) OR AB ( moral OR morals OR morally OR morality OR virtue OR virtues OR conscience OR "professional ethic" OR "professional ethics" OR "clinical ethic" OR "clinical ethics" OR "medical ethic" OR "medical ethics" OR "dental ethic" OR "dental ethics" OR "nursing ethic" OR "nursing ethics" OR "pharmaceutical ethic" OR "pharmaceutical ethics" OR "pharmacy ethic" OR "pharmacy ethics" OR "ethical analysis" OR "ethical analyses" OR "ethical climate" OR "ethical competence" OR "workplace ethic" OR "workplace ethics" OR "organizational ethic" OR "organizational ethics" OR "organisational ethic" OR "organisational ethics" OR "ethical dilemma" OR "ethical dilemmas" OR "ethical difficulty" OR "ethical difficulties" OR "ethical conflict" OR "ethical conflicts" OR "ethical environment" OR "ethical environments" OR "ethical practice" OR "ethical practices" OR "ethical issue" OR "ethical issues" OR "professional code" OR "professional codes" OR "professional value" OR "professional values" ) OR KW ( ethic* OR moral OR morals OR morally OR morality OR virtue OR virtues OR conscience OR "professional ethic" OR "professional ethics" OR "clinical ethic" OR "clinical ethics" OR "medical ethic" OR "medical ethics" OR "dental ethic" OR "dental ethics" OR "nursing ethic" OR "nursing ethics" OR "pharmaceutical ethic" OR "pharmaceutical ethics" OR "pharmacy ethic" OR "pharmacy ethics" OR "ethical analysis" OR "ethical analyses" OR "ethical climate" OR "ethical competence" OR "workplace ethic" OR "workplace ethics" OR "organizational ethic" OR "organizational ethics" OR "organisational ethic" OR "organisational ethics" OR "ethical dilemma" OR "ethical dilemmas" OR "ethical difficulty" OR "ethical difficulties" OR "ethical conflict" OR "ethical conflicts" OR "ethical environment" OR "ethical environments" OR "ethical practice" OR "ethical practices" OR "ethical issue" OR "ethical issues" OR "professional code" OR "professional codes" OR "professional value" OR "professional values" ) OR SU ( ethic* OR moral OR morals OR morally OR morality OR virtue OR virtues OR conscience OR "professional ethic" OR "professional ethics" OR "clinical ethic" OR "clinical ethics" OR "medical ethic" OR "medical ethics" OR "dental ethic" OR "dental ethics" OR "nursing ethic" OR "nursing ethics" OR "pharmaceutical ethic" OR "pharmaceutical ethics" OR "pharmacy ethic" OR "pharmacy ethics" OR "ethical analysis" OR "ethical analyses" OR "ethical climate" OR "ethical competence" OR "workplace ethic" OR "workplace ethics" OR "organizational ethic" OR "organizational ethics" OR "organisational ethic" OR "organisational ethics" OR "ethical dilemma" OR "ethical dilemmas" OR "ethical difficulty" OR "ethical difficulties" OR "ethical conflict" OR "ethical conflicts" OR "ethical environment" OR "ethical environments" OR "ethical practice" OR "ethical practices" OR "ethical issue" OR "ethical issues" OR "professional code" OR "professional codes" OR "professional value" OR "professional values" )

AND

### S2 Healthcare professionals

TI ( "health personnel" OR "healthcare personnel" OR "care personnel" OR "medical personnel" OR "care provider" OR "care providers" OR "healthcare provider" OR "healthcare providers" OR "care worker" OR "care workers" OR "healthcare worker" OR "healthcare workers" OR "health worker" OR "health workers" OR "care professional" OR "care professionals" OR "healthcare professional" OR "healthcare professionals" OR "health professional" OR "health professionals" OR "healthcare team" OR "healthcare teams" OR "care team" OR "care teams" OR "health employee" OR "health employees" OR "hospital staff" OR "medical staff" OR "healthcare staff" OR "care staff" OR "health staff" OR "hospital personnel" OR nurse OR nurses OR "nursing staff" OR "nursing team" OR "nursing teams" OR midwife OR midwives OR physician OR physicians OR allergists OR anesthesiologists OR cardiologists OR dermatologists OR endocrinologists OR gastroenterologists OR "general practitioners" OR geriatricians OR hospitalists OR nephrologists OR neurologists OR oncologists OR ophthalmologists OR otolaryngologists OR pathologists OR pediatricians OR physiatrists OR pulmonologists OR radiologists OR rheumatologists OR surgeons OR urologists OR neonatologists OR neurosurgeons OR psychiatrists OR clinicians OR doctors OR dentists OR anesthetists OR "healthcare practitioner" OR "healthcare practitioners" OR "health practitioner" OR "health practitioners" OR "care practitioner" OR "care practitioners" OR "nursing assistant" OR "nursing assistants" OR "home health aide" OR "home health aides" OR "home care aide" OR "home care aides" OR "healthcare assistants" OR "care assistants" OR "health assistants" OR therapists OR physiotherapists OR dieticians OR dietitians OR nutritionists OR "dental hygienists" OR "dental staff" OR paramedic OR paramedics OR "paramedical personnel" OR "healthcare support workers" OR "social workers" OR psychologists OR psychotherapists OR "psychiatric aides" ) OR AB ( "health personnel" OR "healthcare personnel" OR "care personnel" OR "medical personnel" OR "care provider" OR "care providers" OR "healthcare provider" OR "healthcare providers" OR "care worker" OR "care workers" OR "healthcare worker" OR "healthcare workers" OR "health worker" OR "health workers" OR "care professional" OR "care professionals" OR "healthcare professional" OR "healthcare professionals" OR "health professional" OR "health professionals" OR "healthcare team" OR "healthcare teams" OR "care team" OR "care teams" OR "health employee" OR "health employees" OR "hospital staff" OR "medical staff" OR "healthcare staff" OR "care staff" OR "health staff" OR "hospital personnel" OR nurse OR nurses OR "nursing staff" OR "nursing team" OR "nursing teams" OR midwife OR midwives OR physician OR physicians OR allergists OR anesthesiologists OR cardiologists OR dermatologists OR endocrinologists OR gastroenterologists OR "general practitioners" OR geriatricians OR hospitalists OR nephrologists OR neurologists OR oncologists OR ophthalmologists OR otolaryngologists OR pathologists OR pediatricians OR physiatrists OR pulmonologists OR radiologists OR rheumatologists OR surgeons OR urologists OR neonatologists OR neurosurgeons OR psychiatrists OR clinicians OR doctors OR dentists OR anesthetists OR "healthcare practitioner" OR "healthcare practitioners" OR "health practitioner" OR "health practitioners" OR "care practitioner" OR "care practitioners" OR "nursing assistant" OR "nursing assistants" OR "home health aide" OR "home health aides" OR "home care aide" OR "home care aides" OR "healthcare assistants" OR "care assistants" OR "health assistants" OR therapists OR physiotherapists OR dieticians OR dietitians OR nutritionists OR "dental hygienists" OR "dental staff" OR paramedic OR paramedics OR "paramedical personnel" OR "healthcare support workers" OR "social workers" OR psychologists OR psychotherapists OR "psychiatric aides" ) OR KW ( "health personnel" OR "healthcare personnel" OR "care personnel" OR "medical personnel" OR "care provider" OR "care providers" OR "healthcare provider" OR "healthcare providers" OR "care worker" OR "care workers" OR "healthcare worker" OR "healthcare workers" OR "health worker" OR "health workers" OR "care professional" OR "care professionals" OR "healthcare professional" OR "healthcare professionals" OR "health professional" OR "health professionals" OR "healthcare team" OR "healthcare teams" OR "care team" OR "care teams" OR "health employee" OR "health employees" OR "hospital staff" OR "medical staff" OR "healthcare staff" OR "care staff" OR "health staff" OR "hospital personnel" OR nurse OR nurses OR "nursing staff" OR "nursing team" OR "nursing teams" OR midwife OR midwives OR physician OR physicians OR allergists OR anesthesiologists OR cardiologists OR dermatologists OR endocrinologists OR gastroenterologists OR "general practitioners" OR geriatricians OR hospitalists OR nephrologists OR neurologists OR oncologists OR ophthalmologists OR otolaryngologists OR pathologists OR pediatricians OR physiatrists OR pulmonologists OR radiologists OR rheumatologists OR surgeons OR urologists OR neonatologists OR neurosurgeons OR psychiatrists OR clinicians OR doctors OR dentists OR anesthetists OR "healthcare practitioner" OR "healthcare practitioners" OR "health practitioner" OR "health practitioners" OR "care practitioner" OR "care practitioners" OR "nursing assistant" OR "nursing assistants" OR "home health aide" OR "home health aides" OR "home care aide" OR "home care aides" OR "healthcare assistants" OR "care assistants" OR "health assistants" OR therapists OR physiotherapists OR dieticians OR dietitians OR nutritionists OR "dental hygienists" OR "dental staff" OR paramedic OR paramedics OR "paramedical personnel" OR "healthcare support workers" OR "social workers" OR psychologists OR psychotherapists OR "psychiatric aides" ) OR SU ( "health personnel" OR "healthcare personnel" OR "care personnel" OR "medical personnel" OR "care provider" OR "care providers" OR "healthcare provider" OR "healthcare providers" OR "care worker" OR "care workers" OR "healthcare worker" OR "healthcare workers" OR "health worker" OR "health workers" OR "care professional" OR "care professionals" OR "healthcare professional" OR "healthcare professionals" OR "health professional" OR "health professionals" OR "healthcare team" OR "healthcare teams" OR "care team" OR "care teams" OR "health employee" OR "health employees" OR "hospital staff" OR "medical staff" OR "healthcare staff" OR "care staff" OR "health staff" OR "hospital personnel" OR nurse OR nurses OR "nursing staff" OR "nursing team" OR "nursing teams" OR midwife OR midwives OR physician OR physicians OR allergists OR anesthesiologists OR cardiologists OR dermatologists OR endocrinologists OR gastroenterologists OR "general practitioners" OR geriatricians OR hospitalists OR nephrologists OR neurologists OR oncologists OR ophthalmologists OR otolaryngologists OR pathologists OR pediatricians OR physiatrists OR pulmonologists OR radiologists OR rheumatologists OR surgeons OR urologists OR neonatologists OR neurosurgeons OR psychiatrists OR clinicians OR doctors OR dentists OR anesthetists OR "healthcare practitioner" OR "healthcare practitioners" OR "health practitioner" OR "health practitioners" OR "care practitioner" OR "care practitioners" OR "nursing assistant" OR "nursing assistants" OR "home health aide" OR "home health aides" OR "home care aide" OR "home care aides" OR "healthcare assistants" OR "care assistants" OR "health assistants" OR therapists OR physiotherapists OR dieticians OR dietitians OR nutritionists OR "dental hygienists" OR "dental staff" OR paramedic OR paramedics OR "paramedical personnel" OR "healthcare support workers" OR "social workers" OR psychologists OR psychotherapists OR "psychiatric aides" )

AND

### [S3 Measuring instrument](https://www.ncbi.nlm.nih.gov/mesh/68011795)

TI ( surveys OR survey OR questionnaires OR questionnaire OR instrument OR instruments OR scale OR scales OR screening OR validation OR validity OR validated OR "internal consistency" OR reproducibility OR reliability OR responsiveness OR "ceiling effects" OR "factor analysis" OR "factor analyses" OR psychometric OR psychometrics ) OR AB ( surveys OR survey OR questionnaires OR questionnaire OR instrument OR instruments OR scale OR scales OR screening OR validation OR validity OR validated OR "internal consistency" OR reproducibility OR reliability OR responsiveness OR "ceiling effects" OR "factor analysis" OR "factor analyses" OR psychometric OR psychometrics ) OR KW ( surveys OR survey OR questionnaires OR questionnaire OR instrument OR instruments OR scale OR scales OR screening OR validation OR validity OR validated OR "internal consistency" OR reproducibility OR reliability OR responsiveness OR "ceiling effects" OR "factor analysis" OR "factor analyses" OR psychometric OR psychometrics ) OR SU ( surveys OR survey OR questionnaires OR questionnaire OR instrument OR instruments OR scale OR scales OR screening OR validation OR validity OR validated OR "internal consistency" OR reproducibility OR reliability OR responsiveness OR "ceiling effects" OR "factor analysis" OR "factor analyses" OR psychometric OR psychometrics )

### S4

S1 AND S2 AND S3

Result: 889

### S5 Filter

Limiters: 1994-. Source types: academic journals. Language: english.

Result: 582
