# Supplementary material for: Psychometric properties of instruments measuring ethical climate among healthcare professionals in care settings pre-pandemic: a systematic review
Source: BMC Med Ethics. 2025 Oct 8;26:125. doi: 10.1186/s12910-025-01311-4 (PMC12506347; doi:10.1186/s12910-025-01311-4)
Supplement: Supplementary file 2 — Supplementary Material 2 [file 12910_2025_1311_MOESM2_ESM.docx]

**Supplemental Material 2**

Appendix supplementary 2. Quality criteria for measurement properties (Terwee et al. 2007)

| **Property** | **Definition** |  | **Quality criteria** |
| --- | --- | --- | --- |
| 1. Content validity | The extent to which the domain of interest is comprehensively sampled by the items in the questionnaire | + | A clear description is provided of the measurement aim, the target population, the concepts that are being measured, and the item selection AND target population and (investigators OR experts) were involved in item selection; |
|  |  | ? | A clear description of above-mentioned aspects is lacking OR only target population involved OR doubtful design or method; |
|  |  | – | No target population involvement; |
|  |  | 0 | No information found on target population involvement |
| 2. Internal consistency | The extent to which items in a (sub)scale are intercorrelated, thus measuring the same construct | + | Factor analyses performed on adequate sample size (7 * # items and >100) AND Cronbach’s alpha(s) calculated per dimension AND Cronbach’s alpha(s) between 0.70 and 0.95; |
|  |  | ? | No factor analysis OR doubtful design or method; |
|  |  | – | Cronbach’s alpha(s) <0.70 or >0.95, despite adequate design and method; |
|  |  | 0 | No information found on internal consistency. |
| 3. Criterion validity | The extent to which scores on a particular questionnaire relate to a gold standard | + | Convincing arguments that gold standard is “gold” AND correlation with gold standard >0.70; |
|  |  | ? | No convincing arguments that gold standard is “gold” OR doubtful design or method; |
|  |  | – | Correlation with gold standard <0.70, despite adequate design and method; |
|  |  | 0 | No information found on criterion validity. |
| 4. Construct validity | The extent to which scores on a particular questionnaire relate to other measures in a manner that is consistent with theoretically derived hypotheses concerning the concepts that are being measured | + | Specific hypotheses were formulated AND at least 75% of the results are in accordance with these hypotheses; |
|  |  | ? | Doubtful design or method (e.g., no hypotheses); |
|  |  | – | Less than 75% of hypotheses were confirmed, despite adequate design and methods; |
|  |  | 0 | No information found on construct validity. |
| 5. Reproducibility  5.1. Agreement | The extent to which the scores on repeated measures are close to each other (absolute measurement error) |  |  |
|  |  | + | MIC < SDC OR MIC outside the LOA OR convincing arguments that agreement is acceptable; |
|  |  | ? | Doubtful design or method OR (MIC not defined AND no convincing arguments that agreement is acceptable); |
|  |  | – | MIC > SDC OR MIC equals or inside LOA, despite adequate design and method; |
|  |  | 0 | No information found on agreement. |
| 5.2. Reliability | The extent to which patients can be distinguished from each other, despite measurement errors (relative measurement error) | + | ICC or weighted Kappa ≥ 0.70; |
|  |  | ? | Doubtful design or method (e.g., time interval not mentioned); |
|  |  | – | ICC or weighted Kappa < 0.70, despite adequate design and method; |
|  |  | 0 | No information found on reliability. |
| 6. Responsiveness | The ability of a questionnaire to detect clinically important changes over time | + | SDC or SDC < MIC OR MIC outside the LOA OR RR O 1.96 OR AUC > 0.70; |
|  |  | ? | Doubtful design or method; |
|  |  | – | SDC or SDC > MIC OR MIC equals or inside LOA OR RR ≤ 1.96 OR AUC < 0.70, despite adequate design and methods; |
|  |  | 0 | No information found on responsiveness. |
| 7. Floor and ceiling effects | The number of respondents who achieved the lowest or highest possible score | + | <15% of the respondents achieved the highest or lowest possible scores; |
|  |  | ? | Doubtful design or method; |
|  |  | – | >15% of the respondents achieved the highest or lowest possible scores, despite adequate design and methods; |
|  |  | 0 | No information found on interpretation. |
| 8. Interpretability | The degree to which one can assign qualitative meanings to quantitative scores | + | Mean and SD scores presented of at least four relevant subgroups of patients and MIC defined; |
|  |  | ? | Doubtful design or method OR less than four subgroups OR no MIC defined; |
|  |  | 0 | No information found on interpretation. |

MIC = minimal important change; SDC = smallest detectable change; LOA = limits of agreement; ICC = Intraclass correlation; SD = standard deviation.
^a^ + = positive rating; ? = indeterminate rating; – = negative rating; 0 = no information available.
^b^ Doubtful design or method = lacking of a clear description of the design or methods of the study, sample size smaller than 50 subjects (should be at least 50 in every (subgroup) analysis), or any important methodological weakness in the design or execution of the study.
